# Effects of high-fat and low-calcium diets on the structure of filiform and fungiform tongue papillae in rats

**DOI:** 10.1038/s41598-025-17728-1

**Published:** 2025-09-12

**Authors:** Maha Mohamed Shehata Montaser, Aliaa Abdelmoniem Bedeir Eita

**Affiliations:** 1 Arab Academy for Science, Technology and Maritime Transport- College of Dentistry, Alamein Campus, 17 street, number 314, Smouha, Alexandria, Egypt; 2 Faculty of Dentistry, Alexandria University, 29 Fawzy Moaz Street, Alexandria, Egypt

**Keywords:** High-fat diet, Low calcium diet, Filiform papillae, Fungiform papillae, β-catenin, Cell biology, Diseases, Endocrinology, Medical research, Physiology

## Abstract

This study investigated how high-fat and calcium-deficient diets affect the tongue’s structure and function in Wistar albino rats. These diets can lead to obesity, oxidative stress, and inflammation, which alter salivary gland function, impair taste bud regeneration, and disrupt epithelial integrity partly through changes in β-catenin signaling. The research examined histological changes in e filiform and fungiform papillae and evaluated β-catenin expression in the tongue epithelium. Forty-five male Wistar rats albino rats were divided into three groups: control, high-fat diet (HFD), and low calcium diet (LCD). The feeding period lasted 12 weeks. Tongue tissues were collected for histological examination using H&E staining, scanning electron microscopy (SEM), and β-catenin immunohistochemistry. Blood samples were analyzed for calcium, insulin, glucose, cholesterol, HDL, LDL, and triglycerides. Both high-fat and calcium-deficient diets led to significant weight gain, increased insulin levels, and disrupted lipid profiles in rats. The high-fat diet caused moderate damage to histological tongue structures and reduced β-catenin expression. In contrast, the calcium-deficient diet resulted in more severe epithelial damage, complete papillary atrophy, and a significant decrease in β-catenin expression (*p* < 0.001). Both high-fat and calcium-deficient diets adversely affect the filiform and fungiform structure and function, with calcium deficiency showing a more severe and direct impact through disruption of β-catenin signaling and epithelial integrity.

## Introduction

Both a high-fat diet and calcium deficiency can negatively impact taste sensitivity and the structure of tongue papillae. Monitoring dietary components and addressing nutritional deficiencies may assist dietitians and patients in optimizing nutritional plans by regulating satiety.

Moreover, these nutritional factors may serve as risk indicators for oral conditions such as xerostomia and contribute to patient-reported complaints, including altered taste perception. Notably, a previous study showed that chronic low-grade inflammation induced by a high-fat diet decreases the quantity of taste buds by disrupting the balance between cell renewal and death, potentially explaining the taste dysfunction observed in obese individuals^1^.

High-fat diet-induced obesity causes widespread inflammation, which in turn leads to damage to cellular components (DNA, lipids, and proteins). In the context of such a status, disruption of cellular processes was reported, including gene expression, signal transduction, cell development, and apoptosis^2–4^.

It was found that a high-fat diet can cause structural changes to taste buds and tongue papillae, including atrophy and/or reduction in their number. Also, it can result in disrupting the essential pathways for taste cell regeneration, hindering their ability to repair. Moreover, it was suggested that obesity from a high-fat diet causes salivary gland destruction. Xerostomia (dry mouth) from such damage was found to cause structural mucosal changes, including changes in the dorsal tongue surface^5,6^.

Abdel Moneim RA et al. induced diabetes in rats using a high-fat diet and observed significant damage to the filiform papillae, including loss and destruction of their structure. Cellular changes like hyperplasia, degeneration, and nuclear abnormalities were likely caused by excessive reactive oxygen species, leading to disrupted cell membrane permeability. Additionally, there was evidence of inflammation, with infiltrating inflammatory cells and engorged blood vessels^7^.

Calcium is the most abundant mineral in the human body. It is vital for bone health, cell function, and nerve signal transmission^8,9^. It was found that calcium is crucial in regulating taste bud cell renewal by modulating key signaling pathways such as Wnt/β-catenin and Sonic Hedgehog (SHH). By affecting these pathways, calcium aids in the proliferation, differentiation, and maintenance of taste cells, ensuring the continuous renewal and proper function of taste buds. This occurs through influencing the growth and regeneration of basal cells, which mature into functional taste cells. This highlights the significance of calcium in preserving healthy taste perception^10^. Moreover, calcium deficiency was reported to contribute to salivary gland dysfunction^11^.

β-catenin was used as a key marker to assess the integrity of adherens junctions, given its central role in maintaining cell–cell adhesion through its interaction with E-cadherin. Alterations in β-catenin localization or expression can reflect disruptions in junctional stability, particularly under conditions of calcium deficiency, which is known to impair adherens junction structure^12^.

Despite these findings, current data on the interplay between dietary imbalances and gustatory function remains limited, highlighting the need for comprehensive investigations to elucidate these mechanisms and their clinical implications further.

The present study aimed to evaluate the effects of an induced high-fat and low-calcium diet on the histological features of filiform and fungiform papillae in albino rats. Moreover, it was to evaluate the immunohistochemical expression of beta-catenin in tongue epithelial cells.

## Results

### Body weight and serum parameters

After 12 weeks, both the high-fat diet (HFD) and low calcium diet (LCD) exhibited increased body weight and elevated serum insulin levels compared to the control group, indicating the development of insulin resistance. The LCD group had reduced serum calcium levels, confirming effective calcium depletion, while the HFD group showed a slight, non-significant change in calcium. Total cholesterol and HDL (high-density lipoproteins) levels increased in both experimental groups, with LDL(low-density lipoproteins) levels elevated in the LCD group. Triglyceride levels increased in the HFD group, whereas a slight reduction was observed in the LCD group without statistical significance. Although glucose levels were higher in the HFD group, this change did not reach significance, suggesting a possible prediabetic tendency. These findings indicate that both diets disrupted metabolic and lipid profiles in distinct ways: the HFD was more strongly associated with hyperlipidemia, while the LCD primarily affected calcium homeostasis and LDL levels. (Table 1)

**Table 1:** Body weight and serum parameters of the three study groups after 12 weeks (Mean ± SD).

	HFD group	LCD group	Control group	P1	P2	P3
Body weight (gm)	181.9875 ± 11	195.55 ± 21	168.825 ± 12	0.043*	0.00759*	0.129
Calcium mg/dl	10.7 ± 2	7.5 ± 0.488	9.64 ± 0.32	0.1988	0.0062*	0.028*
Insulin (ng/ml)	32.9 ± 4	27 ± 1.5	22.7 ± 2	0.00114*	0.000761*	0.00165*
Glucose (mg/dl)	118.5 ± 54.6	92.4 ± 13.9	87 ± 10	**0.090**	**0.3388**	**0.1606**
Total cholesterol (mg/dl)	75.8 ± 19	71.5 ± 8.6	51.73 ± 3.5	0.034*	0.012*	**0.418**
LDL (mg/dl)	21.8 ± 18	31.017 ± 5.69	16.9 ± 5.25	**0.29**	**0.02**	**0.2477**
HDL (mg/dl)	40.35 ± 5.39	30.67 ± 1.07	24 ± 1.839	0.0028*	0.004*	0.012*
Triglycerides (mg/dl)	68 ± 8.86	49.5 ± 17	53.84 ± 8.85	0.042*	**0.37**	**0.1180**

HFD: high-fat diet, *: significant (*p* < 0.05), SD: standard deviation, gm: grams, LDL: low-density lipoprotein, HDL: High-density lipoprotein, TG: Triglyceride, mg = milligram, ng = nanogram, ml = millilitre.

p1: p-value for comparing Control and high-fat diet groups.

p2: p-value for comparing between Control and calcium-deficient diet groups.

p3: p-value for comparing between high-fat diet group and calcium calcium-deficient diet group.

*: Statistically significant at *p* ≤ 0.05.

### Histopathological results of the tongue papillae through the light microscope

**Fig. 1:**
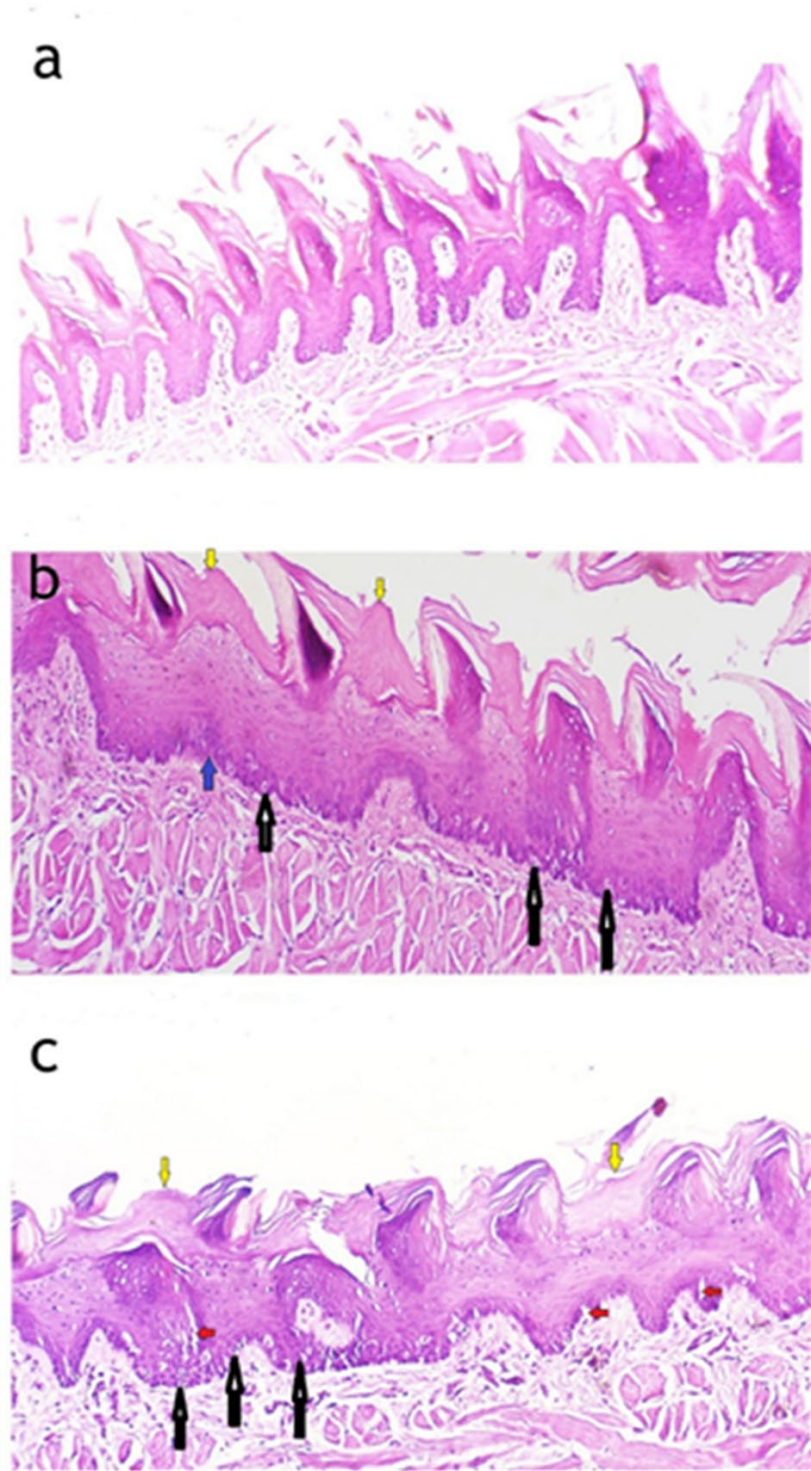
The differences in the filiform appearance between the control group, high-fat diet group, and low calcium diet group. Light micrograph (LM) of filiform papillae on the dorsal tongue surface: (**a**) Control group showing normal epithelial structure. (**b**) High-fat diet group displaying basal cell hyperplasia (blue arrow), the presence of clear cells (black arrows), and areas of hyperkeratosis (yellow arrows) over some papillae. (**c**) Low-calcium diet group demonstrating hyperkeratosis (yellow arrows) and clear cells (black arrows), together with extensive cytoplasmic vacuolations (red arrows). (Hematoxylin & Eosin stain, 100× magnification).

**Fig. 2:**
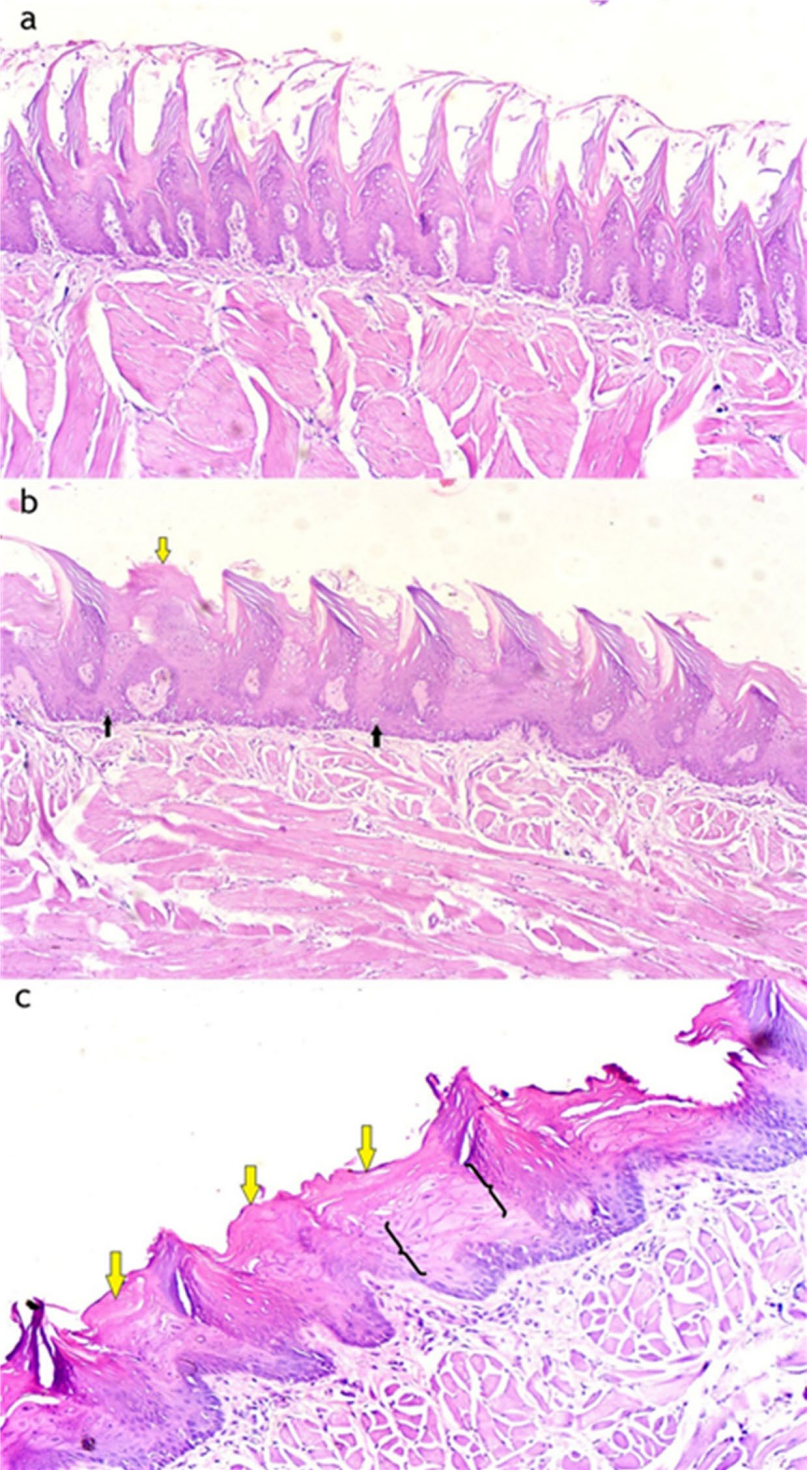
The histological differences in the epithelial layers of the filiform papillae for both the low calcium diet and high-fat diet groups in comparison with the control group. Light micrographs of the epithelial layers of the filiform papillae: (**a**) Control group showing normal epithelial architecture. (**b**) High-fat diet group showing areas of hyperkeratosis (yellow arrow) and the presence of clear basal cells (black arrows). (**c**) Low-calcium diet group showing pronounced hyperkeratosis (yellow arrows) along with hypertrophy of both the granular and prickle cell layers (curved brackets). (Hematoxylin & Eosin stain, 100× magnification).

Light micrographs (LM) of the filiform papillae on the dorsal surface of the tongue reveal notable histological differences among the experimental groups (Figs. 1 and 2). In the control group, the filiform papillae maintain their normal cone-shaped appearance, lined with keratinized epithelium and with well-preserved connective tissue papillae (Figs. 1a and 2a). In the high-fat diet group, the papillae appear with architectural distortion with hyperkeratosis, mild basal cell hyperplasia, and dispersed clear cells are observed within the epithelial layers, and a complete loss of epithelial ridges (Figs. 1b and 2b). In the Low calcium diet group, the filiform papillae show a complete loss of their typical conical shape, with severe hyperkeratinization and areas of loss of epithelial ridges. There is evident hypertrophy in both the granular and prickle cell layers. Numerous clear cells with extensive cytoplasmic vacuolation, which represent degenerative cytoplasmic changes (Figs. 1c and 2c).

**Fig. 3:**
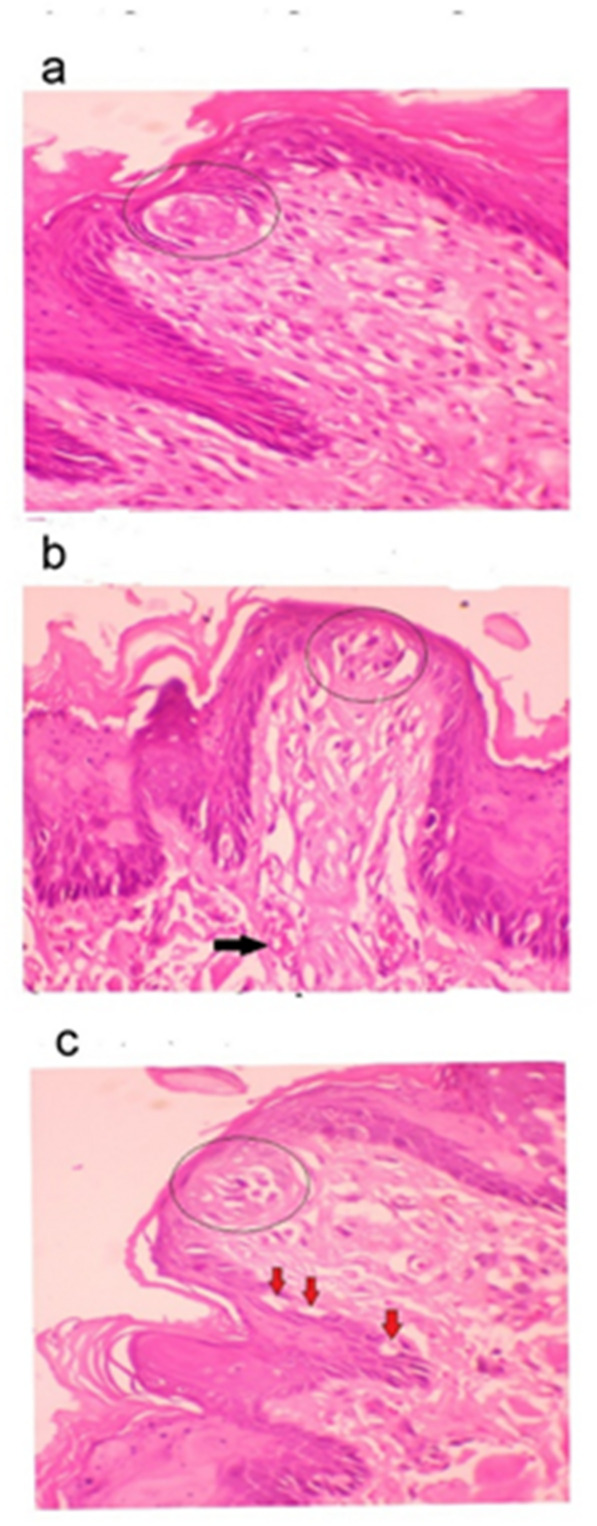
The differences in the fungiform papillae in the three study groups: Light micrograph of the fungiform papillae: (**a**) Control group showing a normal fungiform papilla with a barrel-shaped taste bud (circle). (**b**) High-fat diet group showing a fungiform papilla with separations in the taste buds (circle). A congested blood vessel is also visible (black arrow). (**c**) Low-calcium diet group showing a fungiform papilla with taste buds containing vacuolated and degenerating cells (circle). Cytoplasmic vacuolations are also observed in the epithelial layers (red arrows). (Hematoxylin & Eosin stain, 400× magnification).

Light micrographs of the fungiform papillae demonstrate distinct histological features across the study groups. In the control group (Fig. 3a), the papillae are lined with keratinized epithelium and display a typical barrel-shaped taste bud on the apical surface, with normal cellular morphology. In the high-fat diet group (Fig. 3b), the fungiform papilla retains its general shape; however, the taste bud cells appear separated from each other, and the lamina propria shows signs of dilated and congested blood vessels. In the low calcium diet group (Fig. 3c), the taste buds exhibit severe degenerative cells, and some cytoplasmic vacuolations in the epithelial layers represent apoptotic cells.

### Histomorphometry results

**Fig. 4:**
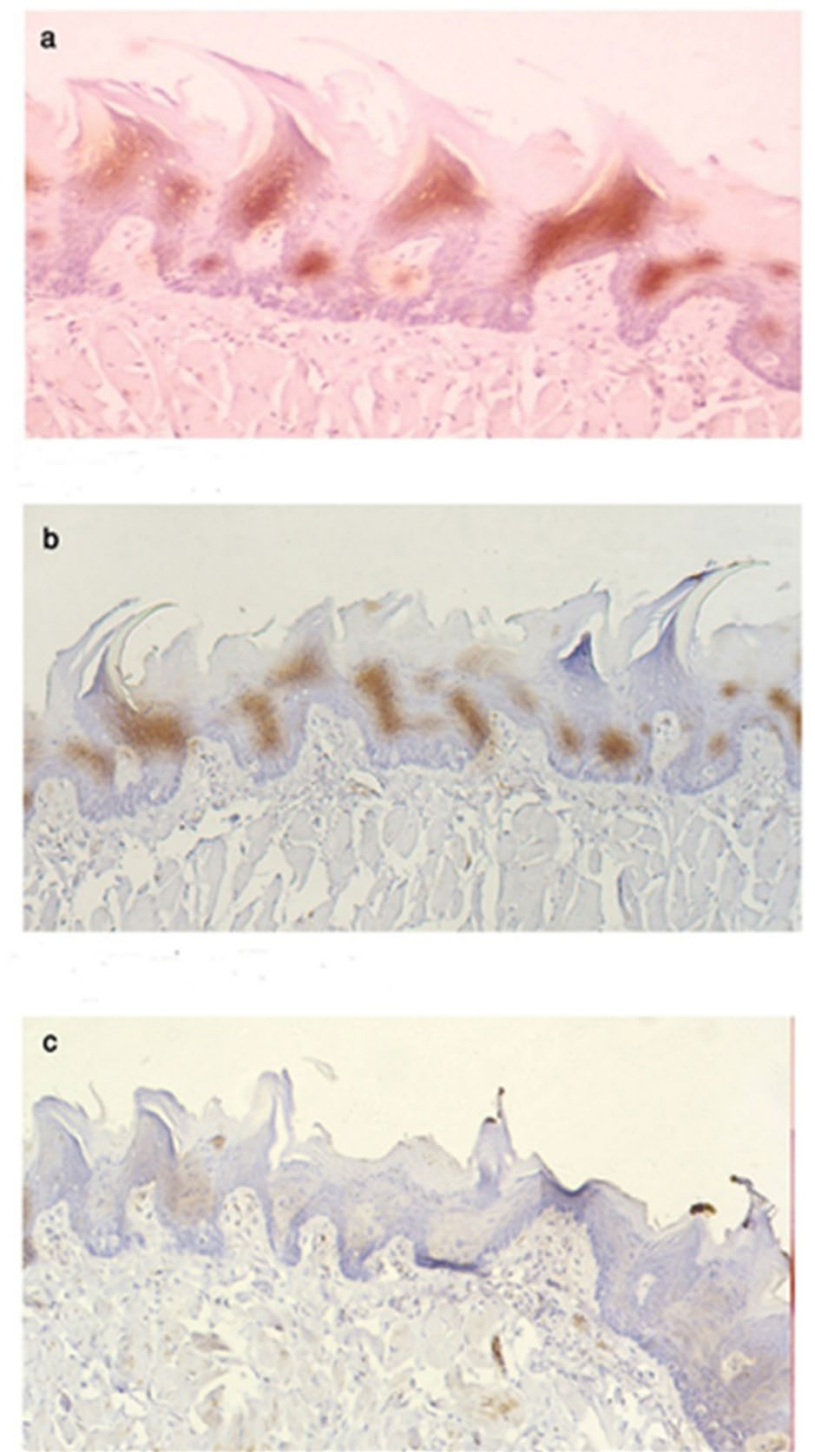
Showing differences between the three groups of β-catenin-immunostained sections. Light microscopy (LM) images of β-catenin immunostaining in the tongue papillae:100x magnification. **(a)** Control group (Group I) shows strong positive immunostaining for β-catenin in the tongue papillae. **(b)** High-fat diet group (Group II) displays mild immunostaining in the tongue papillae.**(c)** Low-calcium diet group (Group III) exhibits minimal to no immunostaining in the filiform papillae.

Quantitative analysis of β-catenin immunohistochemistry revealed a marked reduction in expression levels in both experimental groups compared to the control. The percentage area of β-catenin-positive cells was 25.8% in the control group (Group I), 15.1% in the high-fat diet group (Group II), and 2.59% in the low calcium diet group (Group III) (p₁, p₂, p₃ < 0.001; Table 2). Optical density measurements showed a similar trend, with the highest expression in the control group and the lowest in the low calcium diet group (*p* < 0.05; Table 3). These findings confirm that both diets reduced β-catenin expression, with the low calcium diet having the strongest suppressive effect (Fig. 4).

**Table 2:** Comparison between the three studied groups regarding the area percentage of β-catenin immunopositive cells.

Names of the groups	Control group	High-fat diet group	Low calcium diet group	*P*-value P1 (Control vs. HFD)	*P*-value P2 (Control vs. LCD)	*p*-value P3 HFD vs. LCD)
Area⸓ of cells(mean ± SD)	25.8 ± 2.78	15.1 ± 2.3	2.594 ± 1.1	P_1_^*^_4.46 × 10_^–12^	P_2_^***^ 8.07 × 10^–23^	P_3_^**^ _1.93 × 10_^–17^
MIN-MAX	22–30	11–19	1–5			
Median	25.5	15	2.5			

p: p-value for comparing the three studied groups.

p1: p-value for comparing the Control and high-fat diet groups.

p2: p-value for comparing the Control and calcium-deficient diet groups.

p3: p-value for comparing the high-fat diet group and calcium calcium-deficient diet group.

*: Statistically significant at *p* ≤ 0.05.

SD = standard deviation, min = minimum, max = maximum.

**Table 3:** Comparison between the three studied groups regarding the optical density of β-catenin immunopositive cells.

Names of the groups	Control group	High-fat diet group	Low calcium diet group	*P*-value P1 (Control vs. HFD)	*P*-value P2 (Control vs. LCD)	*p*-value P3 HFD vs. LCD)
Optical density of β-catenin (mean ± SD)	0.218 ± 0.009	0.088 ± 0.011	0.019 ± 0.016	P_1_^**^ 7.70 × 10^–25^	P_2_^***^ 5.31 × 10^–27^	P_3_^*^ _6.70 × 10_^–14^
MIN-MAX	0.202- 0.230	0.070 - 0.106	0- 0.045			

p: p-value for comparing the three studied groups.

p1: p-value for comparing the Control and high-fat diet groups.

p2: p-value for comparing between Control and calcium-deficient diet groups.

p3: p-value for comparing between high-fat diet group and calcium calcium-deficient diet group.

*: Statistically significant at *p* ≤ 0.05.

SD = standard deviation, min = minimum, max = maximum.

### Scanning electron microscopy results

#### Control group

**Fig. 5:**
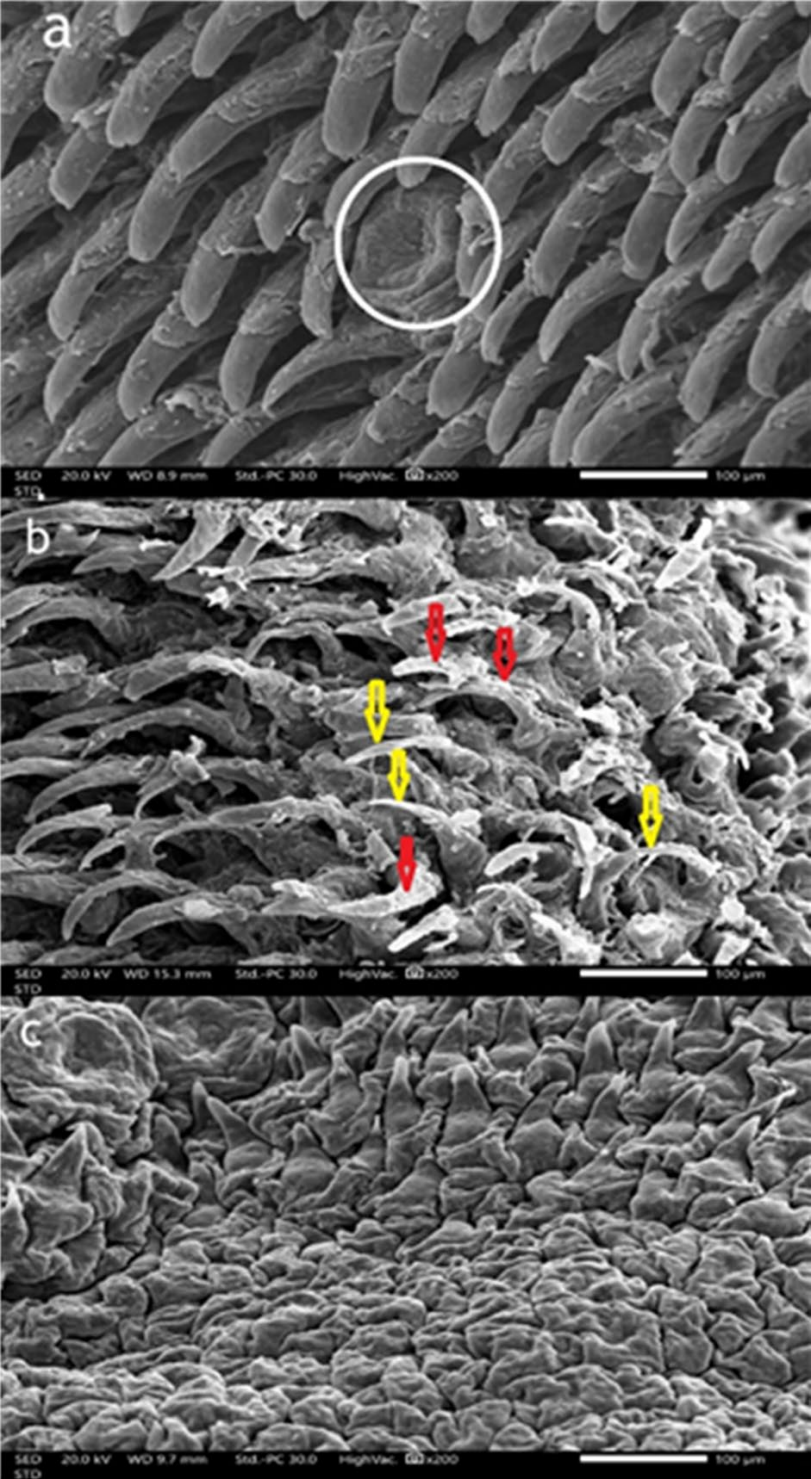
Scanning electron micrographs to compare the filiform papillae on the dorsal tongue surface for the three groups. Scanning electron micrographs of filiform papillae on the dorsal surface of the tongue. (**a**) Control group showing densely arranged filiform papillae oriented anteroposteriorly, with a well-defined fungiform papilla between them (circle). (**b**) High-fat diet group showing irregular, thinned filiform papillae (yellow arrows) with epithelial desquamation (red arrows). (**c**) Low-calcium diet group showing severely atrophic, blunted papillae with a rough, disorganized surface. Scale bar = 100 μm; magnification ×200.

**Fig. 6:**
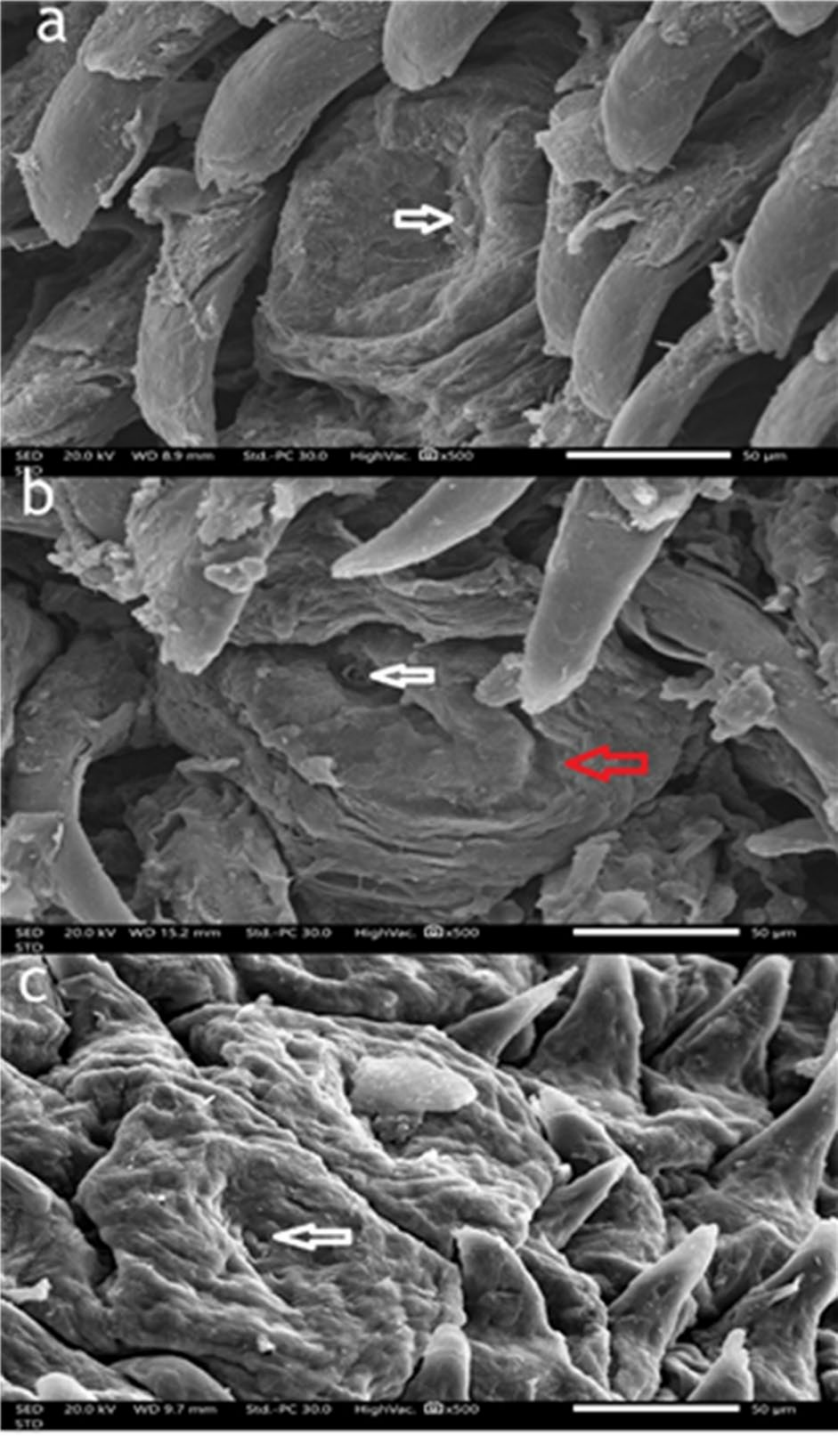
Scanning electron micrograph to compare the fungiform papillae on the dorsal surface of the tongue for the three groups. Scanning electron micrographs of fungiform papillae on the dorsal surface of the tongue. (**a**) Control group showing intact fungiform papillae with a well-defined gustatory pore (white arrow). (**b**) High-fat diet group showing surface irregularities with partial erosion (red arrow) and an ill-defined taste pore (white arrow). (**c**) Low-calcium diet group showing severely atrophic, flattened fungiform papillae with a highly irregular, rough surface. An ill-defined taste pore is also noted (white arrow). Scale bar = 50 μm; magnification ×500.

The dorsal surface of the tongue in the control group displayed a dense, orderly distribution of filiform papillae. These papillae were elongated, slender, and tightly packed, with well-defined keratinized tips, indicating a healthy epithelial surface and intact structural integrity. Scanning electron microscopy further confirmed this, showing sharply pointed, regularly aligned, and densely distributed filiform papillae with distinct keratinization—hallmarks of a functionally intact tongue surface (Fig. 5a). At higher magnification (×500), the fungiform papillae appeared well-developed with dome-shaped, smooth surfaces with normal taste pore and the filiform papillae were surrounded by uniformly arranged filiform papillae, signifying a healthy lingual mucosa and preserved taste function (Fig. 6a).

#### High-Fat diet group

In the high-fat diet group, notable morphological alterations were evident. The filiform papillae appeared irregular, disorganized, and less dense compared to the control group. SEM imaging revealed thin and broken filiform structures with an overall disorganized surface and decreased papillary density, indicating diet-induced degeneration (Fig. 5b). Additionally, the fungiform papillae displayed moderate morphological disruption, including surface roughness, partial erosion, and poorly defined taste pores. Surrounding filiform papillae were irregular and fragmented, suggesting that a high-fat diet contributes to early degenerative changes and compromises gustatory tongue functions (Fig. 6b).

#### Low calcium diet group

The calcium-deficient diet group exhibited the most severe morphological deterioration. The filiform papillae were either markedly atrophic or absent, leaving a flattened, smooth, and irregular epithelial surface. SEM analysis revealed blunt, shortened, or flattened papillae (Fig. 5c). At higher magnification, fungiform papillae were extensively flattened and distorted, with poorly defined taste pore. These extensive changes suggest that calcium deficiency severely impairs the maintenance and regeneration of both filiform and fungiform papillae. (Fig. 6c)

## Discussion

A high-fat diet leads to obesity, which triggers systemic inflammationresulting in cellular damage. This disrupts key cellular functions such as gene expression and cell development. Structurally^2–4^it harms the tongue by reducing and impairing the regeneration of taste buds and papillae, while also damaging salivary glands, leading to dry mouth (xerostomia) and mucosal changes^1,5,6^. Calcium is vital for many physiological functions, including taste bud renewal through pathways like Wnt/β-catenin and SHH^10^. Its deficiency can impair salivary gland function^11^.

Considering the harmful effects of high-fat and low-calcium diets on overall and oral health, this study aimed to compare their impact on the tongue’s histological structure and assess beta-catenin expression in the tongue epithelium of albino rats.

The FDA provides guidelines for using rats in obesity-related preclinical studies, with male rats chosen to avoid hormonal interference^13^. Studies show that 12 weeks on a high-fat or low calcium diets induces obesity and insulin resistance in rats, which can lead to histological changes in the tongue’s specialized mucosa^14^.

This study found that both high-fat and low-calcium diets led to significant weight gain in rats compared to the control group. High-fat diets promote weight gain by impairing fat oxidation and increasing fat storage^15^. Similarly, calcium deficiency increases levels of 1,25-dihydroxyvitamin D and parathyroid hormone(PTH), raising intracellular calcium in fat cells, which enhances fat storage and reduces fat breakdown^16^.

The study found that rats on a low calcium diet had significantly lower serum calcium levels compared to the control and high-fat diet groups, confirming that dietary calcium directly influences blood calcium levels^17^. In contrast, the high-fat diet group showed a slight, non-significant increase in serum calcium. Also, this study found that both low calcium diet and high-fat diets significantly increased blood insulin levels, aligning with Mitri et al.^18^who highlighted calcium’s role in supporting insulin sensitivity. Kołodziej U et al.^2^ and Zalewska et al.^19^ further linked elevated free fatty acids from high-fat diets to insulin resistance. Although blood glucose levels rose slightly in the experimental groups, the increase was not significant, reflecting a prediabetic state. These findings are supported by M. Montaser et al.^20^ and Sánchez M et al.^21^who reported similar effects of diet-induced insulin resistance and the benefits of calcium supplementation, which increase insulin sensitivity.

This study found that a low calcium diet significantly increased serum cholesterol levels, supporting Reid et al. (2002)^22^who proposed that calcium reduces cholesterol by binding bile acids and promoting their excretion. Similarly, Ghasi et al. (2000)^23^reported elevated cholesterol in rats on a high-fat diet. Both high-fat and low calcium diets also led to significant increases in HDL and LDL levels, aligning with Jacqmain et al. (2003)^24^who linked low calcium intake to altered lipid metabolism and increased body fat. Triglyceride (TG) levels rose significantly in the high-fat group, consistent with Schrauwen et al. (2000)^25^who noted increased TGs due to enhanced fat oxidation and reduced glycogen storage. In contrast, the low calcium diet group showed a slight, non-significant TG decrease, possibly due to increased TG clearance via hepatic receptors^26^.

Consistent with previous studies by Khalil, NM^27^and Abdel Moneim, RA^7^the current findings showed basal cell hyperplasia and loss of epithelial ridges in filiform papillae. These changes are linked to hyperglycemia as high-fat diet-induced insulin resistance, which disrupts growth factor signaling (IGF-1, EGF), impairing basal cell proliferation and leading to a flattened epithelial–connective tissue interface. While others display basal cell hyperplasia as a compensatory reaction to localized cellular injury or inflammation^28^.

H&E staining revealed an increase in clear cells with distinct nuclei and cytoplasmic borders. These are most likely non-keratinocyte inflammatory cells infiltrating the epithelium, which reflects the chronic low-grade inflammatory state induced by obesity. This finding aligns with previous reports linking high-fat diets to oxidative stress and inflammatory cell recruitment in oral tissues^29^. Moreover, the lamina propria of fungiform papillae in the high-fat diet group showed dilated, congested blood vessels, likely indicating an inflammatory response to facilitate immune cell infiltration. This is in line with Kaufman, A. et al.(2018), who indicate that HFD mice for 8 weeks leads to inflammatory responses^1^.

Significant hyperkeratosis was observed in H&E sections. Similar findings by Rodgers^30^ and Akai^31^ linked increased keratin gene expression in diabetic mice. This upregulation was associated with enhanced epithelial cell differentiation, which they suggested was driven by calcium signaling pathways activated under high-glucose conditions.

Although histological analysis revealed cellular and structural changes, SEM provided complementary surface-level details such as papillary shape, keratinization, and epithelial integrity. These three-dimensional features, not visible under light microscopy, offered additional evidence of tissue degeneration and enhanced the interpretation of the functional impact of dietary interventions on tongue morphology. The High-fat diet group shows irregular, very thin, with desquamation of their epithelial covering which enhances the degenerative changes caused by obesity.

Histological and ultrastructural analysis of the high-fat diet group revealed degenerative changes in fungiform taste buds, including shrunken and poorly defined gustatory pores. These findings align with Kaufman et al. (2018), who demonstrated that mice fed a high-fat diet for eight weeks became obese and exhibited a significant reduction in taste bud numbers compared to those on a healthy diet, attributing this to obesity-induced inflammation that inhibits taste bud renewal^1^.

Furthermore, Takai et al. (2019) found that insulin signaling plays a crucial role in taste cell proliferation and differentiation, suggesting that disruptions in insulin pathways may impair taste bud maintenance. Therefore, the serological evidence of insulin resistance observed in our study may contribute to the degeneration of taste buds in high-fat diet-fed rats^32^.

The low calcium diet group exhibited more severe taste bud degeneration and recessed taste pores than the high-fat diet group, as seen in both light and electron micrographs. This is because calcium directly supports taste bud renewal and proliferation by modulating key signaling pathways like Wnt/β-catenin and SHH. Through its influence on G-Protein-Coupled Receptors(GPCRs), calmodulin, and transcription factors, calcium ensures the stabilization of β-catenin and activation of glioma-associated oncogene(GLI)proteins, which are vital for maintaining and differentiating basal taste cells^33^.

In the low calcium diet group, the filiform papillae exhibited more pronounced cytoplasmic vacuolations and epithelial deterioration compared to the high-fat diet group. Calcium deficiency disrupts mitochondrial activity and interferes with calcium-dependent protein folding, leading to endoplasmic reticulum (ER) stress and initiation of the unfolded protein response^34,41^ which compromises cell viability and tissue structure This was confirmed by scanning electron microscopy, which revealed significant epithelial degeneration, including noticeable atrophy, surface flattening, and a loss of papillary architecture. As calcium is essential for epithelial adhesion, keratinocyte differentiation, and barrier integrity, its absence weakens desmosomes connections and disturbs vital intracellular signaling pathways, reinforcing calcium’s critical role in preserving epithelial structure in mucosal and skin tissues^35^.

In the low calcium diet group, pronounced hyperkeratosis and hypertrophy of the prickle and granular layers were observed, more so than in the high-fat diet group. This is due to disrupted keratinocyte differentiation, as calcium is essential for cell maturation in the epithelium. Calcium deficiency impairs signaling through Calcium-Sensing Receptor(CaSR) and pathways like Protein Kinase C (PKC), MAPK, and calmodulin, leading to abnormal cell accumulation and expansion of cells in the intermediate layers^36,37^. Additionally, disruption of the normal calcium gradient interferes with keratinization and delays cell shedding, contributing to hyperkeratosis^38^.

For the beta-catenin results, the low calcium diet group showed a significant reduction in β-catenin expression due to impaired cadherin-mediated cell adhesion, which relies on extracellular calcium. This disruption weakens adherens junctions, leading to β-catenin degradation, loss of epithelial cohesion, and tissue degeneration. Compared to high-fat diets, calcium deficiency has a more direct and severe impact on β-catenin loss, as it fundamentally compromises junctional stability^39^. The study by Lickert et al. (2000) supports our use of β-catenin immunohistochemistry to evaluate the effects of calcium signaling deficiency by demonstrating that β-catenin’s interaction with E-cadherin at adherens junctions is calcium-dependent and crucial for maintaining cell-cell adhesion. The research showed that when calcium availability is compromised, the stability of the E-cadherin/β-catenin complex is significantly reduced, leading to weakened intercellular junctions. This directly supports the idea that calcium deficiency has a more immediate structural impact on junction integrity, highlighting β-catenin’s pivotal role in calcium-regulated adherens junction stability^12^.

Whereas in the high-fat diet group, there was mild beta-catenin immunostaining, indicating disrupted junctional stability. Montaser MM previously reported similar findings in rats fed a high-fat diet for 8 weeks, noting separation between acinar cells in the parotid salivary gland. Insulin resistance and resulting hyperinsulinemia are known to downregulate E-cadherin, thereby compromising adherens junctions. These effects are mediated through insulin signaling pathways that influence both E-cadherin stability and cytoskeletal organization^20^.

This study offers valuable insights by comparing the effects of high-fat and low calcium diets on tongue structure using various analytical methods. It effectively links tissue damage to other molecular mechanisms and β-catenin pathway disruption, which aligns well with previous research. It faces some limitations to be considered in future research, such as assessing the combined impact of high-fat and low calcium diets in order to determine whether their interaction produces greater tissue damage than each diet alone. In addition, future studies should explore the specific effects of these diets on taste buds using targeted molecular markers. It is also recommended that upcoming work focus on capturing and quantifying β-catenin expression in both filiform and fungiform papillae for a more comprehensive analysis.

## Materials and methods

### Ethical compliance

All experimental procedures involving animals were reviewed and approved by the Committee for the Care and Use of Laboratory Animals at AASTMT Alamein Dental University (Approval No. 25/2025). The study was conducted in accordance with the National Institutes of Health (NIH) Guide for the Care and Use of Laboratory Animals (8th Edition), the American Veterinary Medical Association (AVMA) Guidelines for the Euthanasia of Animals (2020), and followed the ARRIVE guidelines for reporting animal research^40^.

### Animal housing and allocation

A total of forty-five healthy male Wistar albino rats, each weighing between 150 and 200 g and approximately 4 months old, were acquired from Alexandria University’s Medical Research Institute’s animal facility. All animals were acclimatized for 7 days before the start of the experiment to adapt to the new environment and minimize stress. Individual rats were maintained under controlled conditions, including a 12-hour light/dark cycle, a consistent temperature of 22 °C, and 50% relative humidity. They were housed in standard polypropylene cages with wood shavings used as bedding material, and no more than 3 rats per cage to prevent overcrowding. Environmental enrichment was provided in the form of cardboard tubes and nesting materials, and free access to water and feed^41^.

Using computer-generated random numbers, the rats were randomly divided into three equal groups (15 rats per group): control, high-fat diet, and low calcium diet groups. To minimize bias, outcome assessors (histological and immunohistochemical evaluators) were blinded to the group allocations^41^.

- **Group I (Control group)**: Received a standard rat diet containing normal levels of calcium (1.1–1.3%) and fat (up to 20%) for 12 consecutive weeks^2^.
- **Group II (High-fat diet group)**: Fed a high-fat diet consisting of 60% fat and 1.1–1.3% calcium for 12 weeks^42^.
- **Group III (low calcium diet group)**: Given a diet low in calcium, with calcium levels reduced to 0.1–0.3% compared to the normal range of 1.1–1.3%^43^ and normal percentage of fats up to 20% for 12 weeks.

Diet was obtained from the animal nutrition department at the Faculty of Agriculture, Alexandria University. Throughout the 12-week trial, body weight was tracked and recorded every day.

### Serum analysis

At the end of the 12 weeks, blood samples were collected from anesthetized rats via the tail vein. The samples were centrifuged at 3000 rpm for 15 min to separate the serum, which was then stored at − 80 °C for further analysis. Standard laboratory techniques were used to measure serum levels of total cholesterol, low-density lipoprotein (LDL), high-density lipoprotein (HDL), and triglycerides, all expressed in mg/dl. Fasting insulin concentrations were assessed using rat-specific ELISA kits, with results reported in ng/ml. Serum glucose levels were determined using an automated analyzer, in ng/ml^20^.

### Animal euthanization

All rats were humanely euthanized using a fatal dose of thiopental sodium salt (T0888, Sigma-Aldrich, USA) was administered intraperitoneally at a dose of 150 mg/kg as the primary euthanasia agent. A 2% injectable lidocaine hydrochloride solution was administered separately before the thiopental injection.

Confirmation of death following euthanasia was performed through the observation of cessation of heartbeat, respiratory movements, and corneal reflex, as per ARRIVE and institutional ethical standards. In addition, percutaneous cardiac puncture was used after loss of consciousness to verify the absence of cardiac activity. Lack of syringe movement and failure to aspirate blood confirmed the absence of cardiac output, ensuring complete and humane euthanasia^40^.

After that, the tongues were meticulously cut in half. While the left half was ready for scanning electron microscopy, the right half was treated for light microscopy and immunohistochemical examination^44^.

### Light microscopic examination

After being fixed in 10% neutral buffered formalin, tongue samples were rinsed with distilled water and progressively dehydrated with progressively higher alcohol concentrations. After being cleaned in xylene, the tissues were embedded in paraffin wax. Each paraffin block was divided into longitudinal slices that were 5 μm thick. For histological analysis, these sections were stained with hematoxylin and eosin (H&E)^27^.

### Immunohistochemical examination

Immunohistochemical staining was performed using the standard avidin-biotin complex method (SAB-PO kit; Nichirei Bioscience, Tokyo, Japan). Paraffin-embedded tissue sections were first deparaffinized in xylene, rehydrated through a graded ethanol series, and rinsed under running tap water. For antigen retrieval, sections were heated in 0.01 M citrate buffer (pH 6.0) at 95 °C for 20 min using a water bath, then allowed to cool at room temperature for another 20 min. Sections were then rinsed in phosphate-buffered saline (PBS) (3 × 5 min). To block endogenous peroxidase activity, the sections were incubated in methanol containing 0.3% hydrogen peroxide for 20 min at room temperature, followed by rinsing in PBS (3 × 5 min)^45^.

To minimize non-specific binding, the tissue sections were pre-incubated with 10% normal serum for 30 min at room temperature. Following the removal of excess serum, they were incubated overnight at 4 °C in a humidified chamber with a primary monoclonal mouse anti-β-catenin antibody(clone 15B8, eBioscience, Cat. No. 14-9791-82, Lot No. 2031859, San Diego, CA, USA).

The following day, after rinsing in PBS (3 × 5 min), sections were incubated with a biotinylated anti-mouse IgA + G + M secondary antibody for 30 min at room temperature, followed by PBS washing. A 30-minute incubation with the avidin-biotin-peroxidase complex followed. Immunoreactivity was visualized using 3,3′-diaminobenzidine tetrahydrochloride (DAB) for 8 min, then counterstained with hematoxylin. PBS was used in place of the primary antibody for negative control sections. Immunohistochemical evaluation was performed on ten randomly selected high-power fields (×100 magnification) per Sect.^45^.

### Scanning electron microscopic examination

Mucus was removed from the dorsal surface of the tongue specimens by gently cleaning them with a soft brush while running water. After that, they were fixed for one and a half to two hours in 2.5% glutaraldehyde made in 0.1 M phosphate buffer (pH 7.2). The samples were washed twice for 15 min each in phosphate buffer after fixation. Post-fixation was carried out using osmium tetroxide in the same buffer for approximately 2 h. Any residual osmium was removed with two additional 15-minute buffer rinses. The specimens were then dehydrated through a graded ethanol series (40%, 60%, 80%, 95%, and 100%), spending about 15 min in each concentration, followed by critical point drying. Finally, a scanning electron microscope was used to examine the samples after they had been thinly coated with gold using a sputter coater^27^.

### Histomorphometry analysis

For quantitative immunohistochemical analysis, three tissue sections were selected per specimen at standardized depths. From each section, a single high-resolution image was captured at 100× magnification using a calibrated digital microscope. These images were analyzed using ImageJ software (version 1.46r; NIH, Bethesda, MD, USA) to assess the optical density (OD) of β-catenin immunostaining and the area percentage of immunopositive cells^27^.

Images were first converted to RGB format (Image > Type > RGB), and the “Color Deconvolution” function was used (Image > Color > Color Deconvolution) with the “H DAB” option. The DAB channel was selected, and the threshold was adjusted (Image > Adjust > Threshold) to isolate positively stained regions while minimizing background interference. After thresholding, the binary image was analyzed using “Set Measurements” (selecting “Area Fraction” and “Mean Gray Value”). The resulting data included the percentage of stained areas and the mean intensity.

**Fig. 7:**
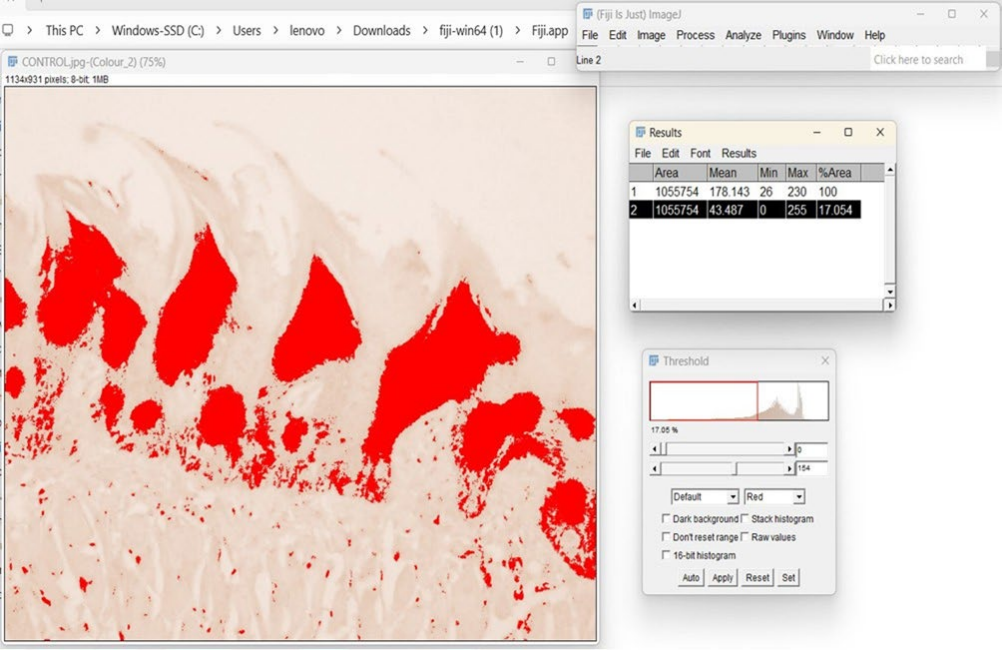
Illustration of the ImageJ analysis method used to quantify the percentage area of β-catenin immunopositive staining, showing threshold adjustment and selection of the stained regions.

Optical density was then calculated using the formula: OD = log₁₀ (255 / mean intensity). For each specimen, average values of OD and area percentage were derived from the three analyzed sections to ensure data reliability and consistency^27^ (Fig. 7).

#### Calculations for sample size

NCSS, LLC, Kaysville, Utah, USA’s Power Analysis and Sample Size Software (PASS 2020) was used to calculate the sample size.45 suitable, healthy male albino rats, each weighing 150–200 grammes and roughly 4 months old (15 per group with a ratio of 1:1), would be the bare minimum of the hypothesised sample size is needed to evaluate the effects of an induced high-fat and low-calcium diet on the histological features of filiform and fungiform papillae in albino rat; taking into consideration an effect size of 30%, significance level of 5%, and power of 80% using Chi square- test. The sample size was estimated using the following formula^46,47^:


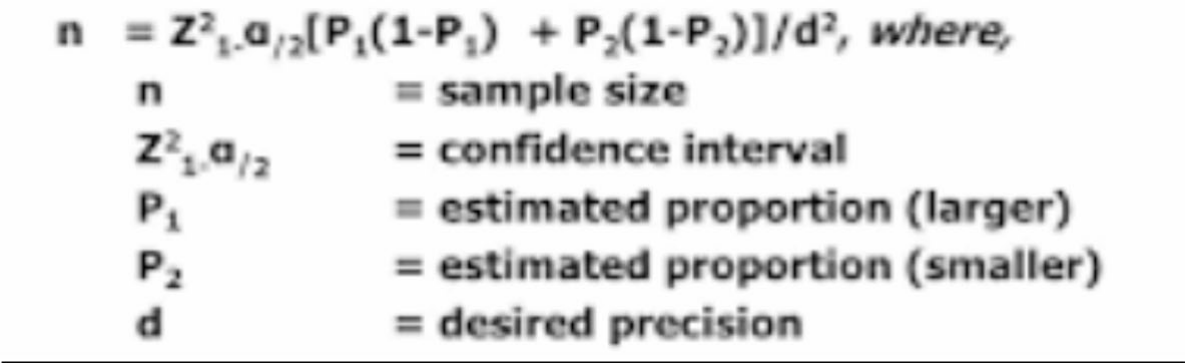


#### Management of information and statistical evaluation

IBM Corp., Armonk, NY, USA’s SPSS version 23.0 for Windows was used for statistical analysis. For every group (*n* = 15), the data were displayed as mean ± standard deviation (SD).One-way analysis of variance (ANOVA) was used to compare means across the three groups (control, high-fat diet, and low calcium diet). Where significant differences were found, Tukey’s post hoc test was applied to identify specific group differences. A significance level of 0.05 was used for all analyses^2^.

## Conclusion

This study demonstrated that both high-fat and low-calcium diets cause distinct structural and molecular changes in the tongues of Wistar albino rats, particularly affecting the integrity of filiform and fungiform papillae. Calcium deficiency had a more pronounced effect, likely due to its essential role in regulating signaling pathways, such as the Wnt/β-catenin pathway, which is involved in cell adhesion and renewal. These findings highlight the critical importance of balanced dietary intake in maintaining oral epithelial health and provide a basis for future research on nutritional influences on taste function and mucosal integrity.

## Data Availability

Data is provided within the manuscript or supplementary information files.
